# Paternal mosaicism for a novel *PBX1* mutation associated with recurrent perinatal death: Phenotypic expansion of the *PBX1*‐related syndrome

**DOI:** 10.1002/ajmg.a.61541

**Published:** 2020-03-06

**Authors:** Peer Arts, Jessica Garland, Alicia B. Byrne, Tristan S.E. Hardy, Milena Babic, Jinghua Feng, Paul Wang, Thuong Ha, Sarah L. King‐Smith, Andreas W. Schreiber, April Crawford, Nick Manton, Lynette Moore, Christopher P. Barnett, Hamish S. Scott

**Affiliations:** ^1^ Genetics and Molecular Pathology Research Laboratory, Centre for Cancer Biology An Alliance Between SA Pathology and the University of South Australia Adelaide South Australia Australia; ^2^ Paediatric and Reproductive Genetics Unit Women's and Children's Hospital Adelaide South Australia Australia; ^3^ School of Pharmacy and Medical Sciences University of South Australia Adelaide South Australia Australia; ^4^ Repromed Dulwich Australia; ^5^ School of Medicine University of Adelaide Adelaide South Australia Australia; ^6^ ACRF Cancer Genomics Facility, Centre for Cancer Biology An Alliance Between SA Pathology and the University of South Australia Adelaide South Australia Australia; ^7^ Australian Genomics Health Alliance Melbourne Victoria Australia; ^8^ School of Biological Sciences University of Adelaide Adelaide South Australia Australia; ^9^ Department of Anatomical Pathology SA Pathology, Women's and Children's Hospital North Adelaide South Australia Australia

**Keywords:** paternal mosaicism, *PBX1*‐related multisystem congenital anomaly syndrome, phenotype expansion, recurrence risk

## Abstract

Autosomal dominant (de novo) mutations in *PBX1* are known to cause congenital abnormalities of the kidney and urinary tract (CAKUT), with or without extra‐renal abnormalities. Using trio exome sequencing, we identified a *PBX1* p.(Arg107Trp) mutation in a deceased one‐day‐old neonate presenting with CAKUT, asplenia, and severe bilateral diaphragmatic thinning and eventration. Further investigation by droplet digital PCR revealed that the mutation had occurred post‐zygotically in the father, with different variant allele frequencies of the mosaic *PBX1* mutation in blood (10%) and sperm (20%). Interestingly, the father had subclinical hydronephrosis in childhood. With an expected recurrence risk of one in five, chorionic villus sampling and prenatal diagnosis for the *PBX1* mutation identified recurrence in a subsequent pregnancy. The family opted to continue the pregnancy and the second affected sibling was stillborn at 35 weeks, presenting with similar severe bilateral diaphragmatic eventration, microsplenia, and complete sex reversal (46, XY female). This study highlights the importance of follow‐up studies for presumed de novo and low‐level mosaic variants and broadens the phenotypic spectrum of developmental abnormalities caused by *PBX1* mutations.

## 1 INTRODUCTION

Congenital anomalies of the kidney and urinary tract (CAKUT) refer to inborn morphogenic defects of the renal system and urinary tract and represent a broad spectrum of clinical manifestations. The large phenotypic heterogeneity of CAKUT ranges from subclinical abnormalities present in the general population, to severe malformations which are sometimes incompatible with life (Knoers & Renkema, 2019; Nicolaou, Renkema, Bongers, Giles, & Knoers, 2015).

De novo mutations in *PBX1* were first identified as a cause of CAKUT in humans in 2017 (MIM: 617641) following earlier work demonstrating renal hypoplasia, renal ectopia, and renal agenesis in a *Pbx1* ^−/−^ mouse model (Heidet et al., 2017; Le Tanno et al., 2017; Schnabel, Selleri, & Cleary, 2003; Slavotinek et al., 2017). The reported heterozygous disease causing mutations in *PBX1* include partial and complete gene deletions, truncating, splice site and missense variants (Figure S1). These de novo variants have been described in individuals with renal hypoplasia/dysplasia and associated urinary tract abnormalities, with a broad range of extra‐renal abnormalities. These include facial dysmorphism, and anatomic abnormalities of the ear, genitalia, heart, and lung. Developmental delay/intellectual disability is common, and growth restriction and hearing loss have also been reported (Table S1; Alankarage et al., 2019; Eozenou et al., 2019; Heidet et al., 2017; Kia, Sarafoglou, Mooganayakanakote Siddappa, & Roberts, 2019; Riedhammer et al., 2017; Slavotinek et al., 2017; Sun et al., 2019; Le Tanno et al., 2017). We present two cases of perinatal death, from independent pregnancies in one family, with a recurrent disease‐causing mutation in *PBX1* due to paternal mosaicism for the mutation.

## 2 METHODS

### 2.1 Patient consent and ethics approval

This study was performed in concordance with the declaration of Helsinki. The family were referred to the clinical genetics unit for consideration of an underlying genetic diagnosis following the first affected pregnancy. The family were counseled and provided consent for enrolment into the Genomic Autopsy Study (HREC/15/WCHN/35, family study ID: PED043), a National Health and Medical Research Council (NHMRC) funded study.

### 2.2 Genomic analysis

Parents‐proband trio whole exome sequencing (WES) was performed on DNA isolated from whole blood. Exonic sequences were enriched from genomic DNA using an Illumina exome capture (38 Mb target) and sequenced at the Genomics Platform of the Broad Institute (Boston, MA). Sequencing reads were aligned to the human reference genome GRCh37/hg19 (BWA 0.7.12) and single nucleotide variants (SNVs) and small insertions/deletions were called using GATK HaplotypeCaller (package version 3.8.0). Copy number variants (CNVs) were detected using an in‐house (unpublished) algorithm. Data from 98 unrelated samples sequenced in the same batch were used to normalize read depth signals before partitioning into bin sizes optimal for the exome capture. Variant filtering was performed to select for rare protein altering variants (SNVs: gnomAD and in‐house frequencies <1% for autosomal recessive [AR] and <0.01% for autosomal dominant [AD], CNVs: no reciprocal overlap with known benign CNVs ≥70%; Lek et al., 2016).

### 2.3 Droplet digital PCR

Given the father's low‐level mosaicism in blood, droplet digital PCR (ddPCR) was performed on a sperm sample to define the recurrence risk. The ddPCR assay for the *PBX1* c.319C > T variant was ordered from Thermo Fisher Scientific (Waltham, MA). The ddPCR assay was performed in triplicate on DNA from four samples (fetal, maternal and paternal blood, and paternal sperm) using a BioRad QX200 instrument.

## 3 RESULTS

### 3.1 Family presentation

**Figure 1:**
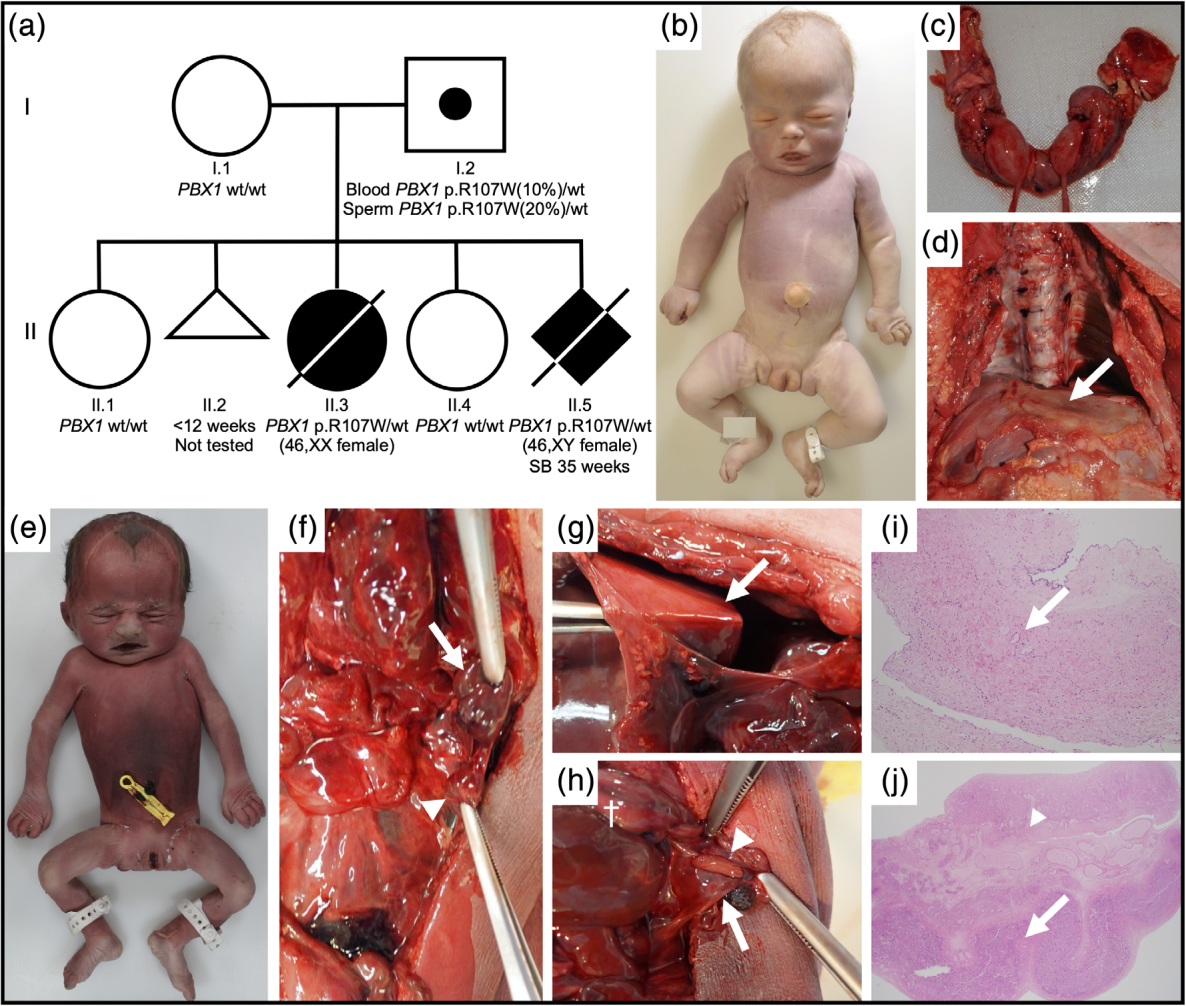
(a) Pedigree, (b–d) Autopsy photographs of the proband (II‐3). (b) Dysmorphic features include hypertelorism, prominent forehead, and downturn corners of the mouth. (c) Horseshoe kidney. (d) Extremely thin, transparent diaphragm (white arrow). (e–j) Autopsy photographs and histology images of the affected sibling (II‐5), (e) Dysmorphic features include deep‐set eyes and prominent forehead. Note normal female genitalia despite 46, XY karyotype. (f) Hypoplastic spleen (white arrow) and tiny accessory spleen (white arrowhead). (g) Paper‐thin diaphragm with forceps visible through diaphragm (white arrow). (h) Macroscopically normal ovary (white arrow), fallopian tube (white arrowhead), and uterus (†). (i) Histology of diaphragm demonstrating complete absence of muscle cells (white arrow). (j) Normal histology of fallopian tube (white arrow) and ovary (white arrowhead)

The proband (II.3) is a one‐day old deceased female infant with syndromic CAKUT (Figure 1a). Her nonconsanguineous healthy parents have no family history of CAKUT. However, the father had an ultrasound in childhood which demonstrated subclinical dilation of one renal pelvis in an otherwise normal urinary tract. He has had no renal‐related illnesses during his adult life.

The couple's firstborn, a 5‐year‐old healthy female, was antenatally diagnosed with an ovarian cyst but did not show any renal anomalies on abdominal ultrasound. Their second pregnancy resulted in a miscarriage before 12 weeks' gestation for which no genetic testing was performed.

The proband was born following their third pregnancy in which a mildly increased nuchal translucency (3.0 mm) and polyhydramnios were noted at 12 weeks' gestation. Early morphology ultrasound (16 weeks) identified hypoplastic lungs, dilated bilateral renal pelves, a horseshoe kidney, small abdominal and chest circumferences, an apparent left‐sided diaphragmatic hernia, and confirmed polyhydramnios. The couple was counseled regarding aneuploidy risk but opted to forego invasive testing and continued the pregnancy. The female infant was born via elective cesarean section at 38 weeks' gestation (birthweight 2,920 g). After delivery, the infant required ventilatory support for severe persistent pulmonary hypertension secondary to pulmonary hypoplasia but deteriorated over 24 hours and died.

Karyotype of the female proband was normal (46, XX) and microarray analysis (Illumina CytoSNP‐850 k) did not identify any CNVs. At autopsy, external examination revealed mildly dysmorphic facial features including hypertelorism, a prominent forehead, and a protuberant tongue (Figure 1b). In addition, overlapping 3–4 fingers (right hand) and 1–2 toes (right foot) were noted. Internal examination showed severe pulmonary hypoplasia associated with a markedly thinned (transparent) diaphragm leading to eventration without true diaphragmatic hernia (Figure 1d). Other findings included asplenia, a horseshoe kidney with bilateral pelvic dilatation, abnormal ureter insertion into the bladder, and a bicornuate uterus (Figure 1c). The placenta was small for gestational age (weight 361 g [NR 2SD 409–589 g]).

Histology showed immature development of the lung, consistent with a congenital alveolar dysplastic pattern. There were thick septa and excessive vessels. Hyaline membranes were seen throughout the lung. Bronchi were correctly positioned and veins were present in the septum, making capillary alveolar dysplasia unlikely. There was marked reduction in the radial alveoli count with some areas showing minimal alveolar tissue between the bronchial and the lung wall. Immunohistochemical stains for cytokeratins and vascular markers were used to further detail the structure of the hypoplastic lungs. The hypoplastic lungs were thought to best represent a congenital alveolar dysplastic pattern. Along with the structural abnormalities, there was a dense neutrophil infiltrate present within the alveoli spaces, consistent with pneumonia.

The fourth pregnancy of the couple was uncomplicated and resulted in the birth of a healthy female who was confirmed negative for the familial *PBX1* mutation.

The couple naturally conceived a fifth pregnancy (II.5). The 12‐week ultrasound scan showed normal nuchal translucency but limited other information. Prenatal diagnosis at 11 weeks' gestation showed a 46, XY karyotype and revealed the presence of the *PBX1* mutation. A 16‐week early morphology ultrasound showed a dilated renal pelvis and possible small diaphragmatic hernia. A follow‐up (20‐week) ultrasound revealed a ventricular septal defect, an overriding aorta, possible pulmonary atresia, bilateral renal pelvis dilatation, and ambiguous genitalia. The diaphragmatic hernia was not confirmed. Decreased fetal movements were noted at 35 weeks and the mother presented with a stillbirth shortly after. Autopsy demonstrated marked thinning/aplasia of the diaphragm leading to eventration, normal female appearing external genitalia with a normally formed vagina, uterus, fallopian tubes and ovaries, a small spleen and smaller accessory spleen, bilaterally small kidneys and bilateral ureteric dilatation, a bell‐shaped chest, and pulmonary hypoplasia (Figure 1e–j). A precise cause of death was not identified at autopsy; however, multiple congenital anomalies and congenital heart disease are both separately associated with an increased risk of stillbirth (Liu et al., 2019; Mcclure et al., 2015). Autopsy identified some subtle placental abnormalities including subchorionitis, early choriodeciduitis, and a small localized infarct but these findings were not considered causative of stillbirth as they are relatively nonspecific common findings. Given the discordant chromosomal (46, XY) and phenotypic sex, the karyotype was checked on a separate sample, and was again confirmed as 46, XY.

### 3.2 Genomic analysis

Filtering for rare, nonsynonymous variants in the proband (II.3) yielded high quality variants affecting one AD and three AR genes (Table S2). Further prioritization for genes associated with renal disorders highlighted one AD de novo variant; a missense variant in *PBX1* (Chr1(hg19):g.164761784C > T; NM_002585.3:c.319C > T;NP_002576.1:p.(Arg107Trp)), that was absent from population databases and not called in either parent. However, manual inspection of sequencing reads showed the variant at low level (7%, 10/136 variant reads; Figure S2) in the paternal blood sample. The *PBX1* p.(Arg107Trp) variant was predicted to be deleterious by multiple computational pathogenicity predictors, such as CADD, SIFT, PolyPhen, and Mutation Taster (Table S3). Follow‐up by ddPCR revealed that the variant was present in 10.4% of paternal blood cells and 20.1% of sperm cells (Figure S3).

## 4 DISCUSSION

*PBX1* encodes the Pre‐B Cell Leukemia Transcription Factor, which regulates the expression of genes involved in tissue development, including morphologic patterning and hematopoiesis (Ficara,Murphy, Lin, & Cleary, 2008; Heidet et al., 2017; Slavotinek et al., 2017; Le Tanno et al., 2017). Highlighting the importance of PBX1 in organogenesis, *Pbx1* ^*−/−*^ mice displaying asplenia, hyposplenia, and diaphragmatic muscularization and tissue patterning defects (Brendolan et al., 2005; Koss et al., 2012; Russell et al., 2012).

Here, we present paternal mosaicism for a *PBX1* mutation leading to perinatal death in two siblings, with the identified variant, p.(Arg107Trp), underlying a severe presentation of the multisystem disorder caused by *PBX1* mutations. The congenital abnormalities observed in our siblings expand the phenotypic spectrum of *PBX1*‐associated disease with unique features including severe diaphragmatic eventration, and asplenia/microsplenia. While unilateral diaphragmatic eventration has been reported in two patients (Slavotinek et al., 2017), our patients' diaphragms were entirely membranous and devoid of muscle tissue.

Genital abnormalities are commonly reported in male patients with *PBX1* mutations (10/14), with the phenotypic spectrum ranging from cryptorchidism to partial development of female internal and/or external genitalia (Alankarage et al., 2019; Eozenou et al., 2019; Kia et al., 2019; Riedhammer et al., 2017; Slavotinek et al., 2017; Le Tanno et al., 2017). Interestingly, the second affected sibling (II‐5; 46, XY) presented here showed complete sex reversal of both internal and external organs, without any signs of male gonad development (no streak gonad). In line with earlier reports (Eozenou et al., 2019; Slavotinek et al., 2017), the phenotypic heterogeneity among patients carrying the same mutation indicates a limited genotype–phenotype correlation, possibly influenced by factors such as gender, genetic modifiers, or skewed gene expression.

In recent years, the contribution of parental mosaicism to de novo mutations has been reported for several (neuro‐)developmental disorders (Acuna‐Hidalgo et al., 2015; Wright et al., 2019). However, potential recurrence of presumed de novo mutations is still not routinely considered in research and diagnostics. Using ddPCR, the low‐level paternal mosaic *PBX1* mutation was detected in 20.1% of sperm cells, indicating an estimated recurrence risk of one in five. For this family, unfortunately, their subsequent affected pregnancy was conceived before these results were available and assisted reproductive options could be considered.

Altogether, we identified recurrence for a novel *PBX1* mutation in two siblings, leading to severe congenital abnormalities and perinatal lethality. The severe diaphragmatic hypoplasia, asplenia/microsplenia and complete sex reversal observed in our patients further expand the clinical phenotypes associated with the PBX1‐related complex multisystem abnormality syndrome. In addition, this report also represents the first mosaic *PBX1* mutation in a parent leading to recurrence, highlighting the relevance of follow‐up screening.

## CONFLICT OF INTEREST

The authors declare no conflict of interest.

## AUTHOR CONTRIBUTIONS

P.A., J.G., A.B.B., C.B. and H.S.S. drafted the manuscript. P.A., M.B. and S.L.K.‐S. coordinated the study. J.F., T.H., P.W. and A.W.S. processed WES data and performed preliminary analyses, and P.A. performed tertiary data analysis. P.A., J.G., A.B.B., T.S.E.H., T.H., S.L.K.‐S., H.S.S. and C.B. contributed to interpretation and discussion of results. A.C., N.M., L.M. performed surgical pathology investigations. C.B. and T.S.E.H. provided clinical care. H.S.S. and C.B. conceived and supervised the study, contributing equally, and should be considered co‐senior authors. All authors read and approved the manuscript.

## Supporting information


**Appendix** **S1**: Supporting information

## Data Availability

Data availability The data presented in this study are available from the corresponding author upon reasonable request.
