# Supplementary material for: Paternal mosaicism for a novel PBX1 mutation associated with recurrent perinatal death: Phenotypic expansion of the PBX1‐related syndrome
Source: Am J Med Genet A. 2020 Mar 6;182(5):1273–7. doi: 10.1002/ajmg.a.61541 (PMC7217179; doi:10.1002/ajmg.a.61541)
Supplement: Supplementary file 1 — Appendix S1: Supporting information [file AJMG-182-1273-s001.docx]

**Supplementary information to**

**Paternal mosaicism for a novel PBX1 mutation associated with recurrent perinatal death: phenotypic expansion of the PBX1-related syndrome**

Peer Arts^1^, Jessica Garland^2^, Alicia B. Byrne^1,3^, Tristan S.E. Hardy^1,4,5^, Milena Babic^1^, Jinghua Feng^3,6^, Paul Wang^6^, Thuong Ha^1^, Sarah L. King-Smith^1,7^, Andreas W. Schreiber^3,6,8^, April Crawford^9^, Nick Manton^9^, Lynette Moore^5,9^, Christopher P. Barnett^2,5,*^, Hamish S. Scott^1,3,5,6,7*^

^1^Genetics and Molecular Pathology Research Laboratory, Centre for Cancer Biology, An alliance between SA Pathology and the University of South Australia, Adelaide, SA, Australia

^2^Paediatric and Reproductive Genetics unit, Women's and Children's Hospital, Adelaide, Australia

^3^School of Pharmacy and Medical Sciences, University of South Australia, Adelaide, Australia

^4^Repromed, Dulwich, South Australia

^5^School of Medicine, University of Adelaide, Adelaide, SA, Australia

^6^ACRF Cancer Genomics Facility, Centre for Cancer Biology, An alliance between SA Pathology and the University of South Australia, Adelaide, SA, Australia

^7^Australian Genomic Health Alliance, Melbourne, VIC, Australia

^8^School of Biological Sciences, University of Adelaide, Adelaide, SA, Australia

^9^Department of Anatomical Pathology, SA Pathology, Women's and Children's Hospital, North Adelaide, Australia

**SUPPLEMENTARY FIGURES**

**Supplementary figure 1** **reported *PBX1* variants**


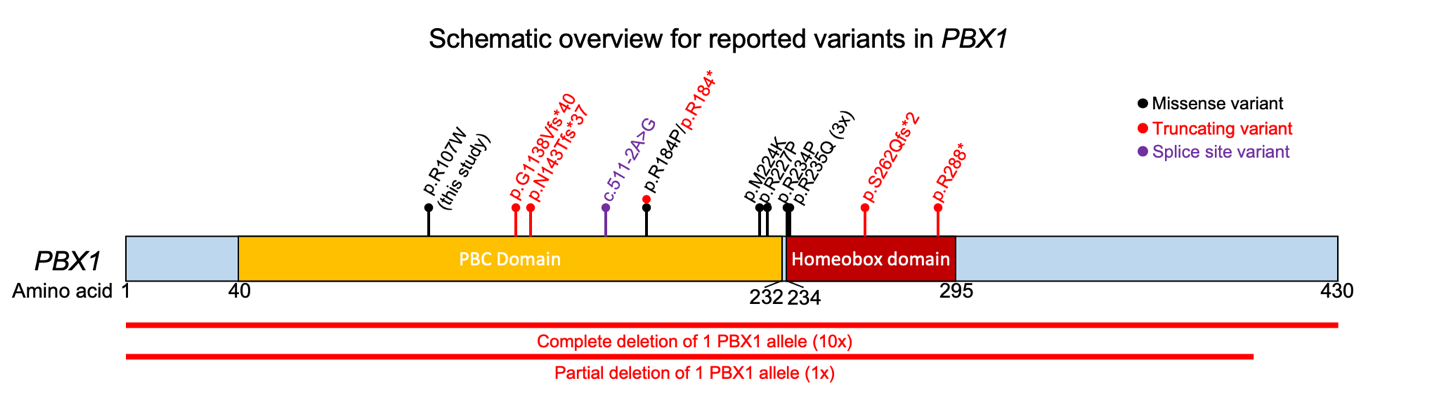


**Supplementary figure 1**: schematic representation of the published variants in *PBX1,* and the variant reported in this study (p.R107W).

**Supplementary figure 2: Exome sequencing data**

**Supplementary figure 2**: IGV screenshot of the region in *PBX1*, showing few paternal (top) reads with the same *PBX1* p.(Arg107Trp) mutation as the proband (bottom).

**Supplementary figure 3 ddPCR data**

**
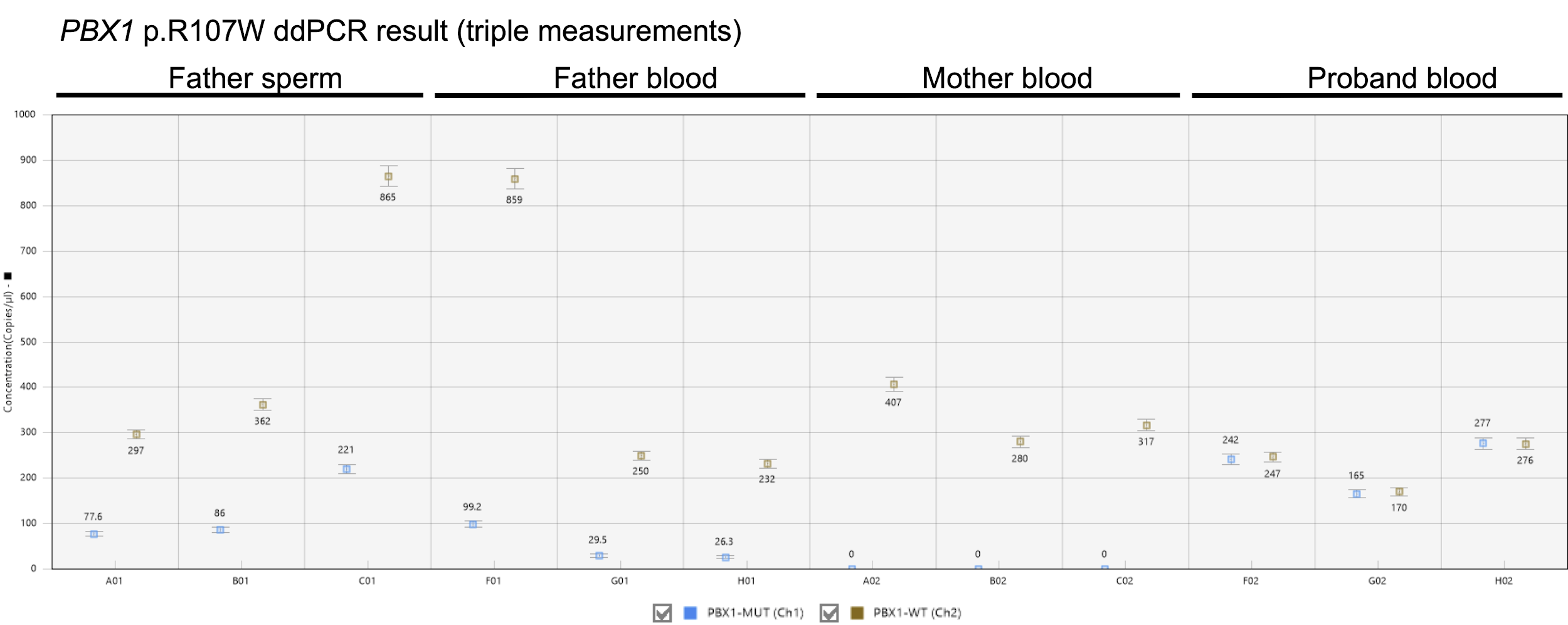
**

**Supplementary figure 3**: Droplet digital PCR results of the *PBX1* mutation in copies of an allele per µl (mutation-blue, wild-type-brown). The allelic ratio is different between paternal sperm and blood, the maternal sample is negative for the mutation, and the proband is heterozygous.

**SUPPLEMENTARY TABLES**

**Supplementary Table 1: The affected organ systems associated with PBX1 mutations reported in literature.**

| **Studies** | **Our patients**  **(n=2)** | **Heidet 2017**  **(n=5)** | **Le Tanno 2017**  **(n=8)** | **Slavotinek 2017 (n=8)** | **Eozenou 2019**  **Riedhammer 2017**  **Sun 2019 (n=3)** |
| --- | --- | --- | --- | --- | --- |
| **Reported PBX1 mutations** | both siblings  p.R107W  (c.319C>T) | 1. p.N143Tfs*37  (c.428delA)  2. p.R184*  (c.550C>T)  3. Splice acceptor exon 4  (c.511-2A>G)  4. Deletion 1 allele of *PBX1*  5. Deletion 1 allele of PBX1 | 1. 6.0-Mb deletion  (chr1:161650414–167622545)  2. 9.2-Mb deletion  (chr1:162703368–171908659)  3. 2.8-Mb deletion  (chr1:163193466 –166058476)  4. 1.5-Mb deletion  (chr1:163574086–165092429)  5. 3.6-Mb deletion  (chr1:163811431–167385298)  6. 0.9-Mb deletion  (chr1:164330973–165207097)  7. 6.9-Mb deletion  (chr1:164501003–171424595)  8. 0.3-Mb deletion  (chr1:164523918–164799811) | 1. p.R184P  (c.551G>C)  2. p.M224K  (c.671T>A)  3. p.R227P  (c.680G>C)  4. p.R234P  (c.701G>C)  5. p.R235Q  (c.704G>A)  6. p.S262Qƒs*2  (c.783dupC)  7. p.R288*  (﻿c.862C>T)  8. p.R235Q  (c.704G>A) | 1. p.G138Vfs*40  (c.413_419del) 2.p.R235Q (c.704G>A) 3. 1.9-Mb deletion (163,444,997-165,316,065) |
| **Inheritance** | paternal mosaic 2/2 | *de novo* 5/5 | *de novo* 8/8 | *de novo* 8/8 | *de novo* 3/3 |
| **Gender** | 1F / 1M^ | 3F / 2M | 4F / 4M | 3F / 5M | 1F^11^ / 2M |
| **Kidney** | 1/2 | 5/5 | 7/8 | 3/8 | 2^8,11^/3 |
| **Urinary tract** | 1/2 | 0/5 | 1/8 | 1/8 | 0/3 |
| **Diaphragm** | 2/2 | nr | nr | 2/8 | 0/3 |
| **Spleen** | 2/2 | nr | nr | nr | nr |
| **Genital** | 2/2 | 0/5 | 3/8 | 5/8 | 2^8,10^/3 |
| **Respiratory** | 2/2 | 0/5 | nr | 2/8 | 0/3 |
| **Cardiovascular** | 1/2 | 0/5 | 4/8 | 5/8 | nr |
| **Brain** | 0/2 | nr | 4/8 | nr | 0/3 |
| **Skeletal** | 0/2 | nr | 3/8 | nr | nr |
| **Craniofacial** | 1/2 | 2/5 | 7/8 | 5/8 | 2^8,10^/3 |
| **Extremities** | 1/2 | 0/5 | 2/8 | 2/8 | 3/3 |
| **Hearing loss** | nr | 2/5 | 2/8 | 1/8 | nr |

F = female, M = male, nr = not reported. ^ Patient is genetically male (46, XY) but phenotypically female.

**Supplementary table 2**

| **Exome sequencing results** | **total Gb** | **mean depth** | **% bases ≥ 20X** | |
| --- | --- | --- | --- | --- |
| Proband (II.3) | 3.9 | 84.2 | 90 | |
| Mother (I.1) | 4.6 | 99.96 | 91.1 | |
| Father (I.2) | 4.3 | 92.58 | 91.3 | |
| **Variant filtering** | **Variant number** |  |  | |
| Proband total variants | 102330 |  |  | |
| **Recessive analysis** |  |  |  | |
| Rare variants (<1% gnomAD & in-house) | 3088 |  |  | |
| Coding, non-synonymous variants | 488 |  |  | |
| Homozygous variants | 0 |  |  | |
| Compound heterozygous variants | 3 | (*CCDC74B*, *FAM186A*, *KIF21A*) | | |
| **Dominant (de novo) analysis** |  |  | |  |
| Extremely rare (<0.001 gnomAD & in-house) | 22 |  | |  |
| Allele depth > 10 | 1 | *PBX1* | | |
| Prioritisation for genes in renal disease | 1 | *PBX1* p.(Arg107Trp) | | |

**Supplementary table 2:** Exome sequencing results and variant filtering overview

**Supplementary table 3**

| **Variant PBX1 p.(Arg107Trp)** | | |
| --- | --- | --- |
| Population frequency | | |
| gnomAD exomes | | 0 (absent) |
| gnomAD genomes | | 0 (absent) |
| gnomAD constraint metrics | | |
| Missense constraint | Z= 3.83 | |
| LoF constraint | pLI = 1 | |
| Pathogenicity predictions | | |
| CADD score (version 1.3) | | 34 |
| SIFT | | Damaging |
| PolyPhen | | Probably damaging |
| Mutation Taster | | Disease causing |
| Conservation | | |
| Gerp score | | 4.56 |

**Supplementary table 3**: Details on population frequencies, evolutionary conservation and pathogenicity predictions for *PBX1* p.(Arg107Trp)

**References in the supplement**

Eozenou, C., Bashamboo, A., Bignon-Topalovic, J., Merel, T., Zwermann, O., Lourenco, D., … Brauner, R. (2019). The TALE homeodomain of PBX1 is involved in human primary testis-determination. *Human Mutation*, *40*(8), 1071–1076. https://doi.org/10.1002/humu.23780

Heidet, L., Morinière, V., Henry, C., De Tomasi, L., Reilly, M. L., Humbert, C., … Jeanpierre, C. (2017). Targeted Exome Sequencing Identifies PBX1 as Involved in Monogenic Congenital Anomalies of the Kidney and Urinary Tract . *Journal of the American Society of Nephrology*, *28*(10), 2901–2914. https://doi.org/10.1681/asn.2017010043

Riedhammer, K. M., Siegel, C., Alhaddad, B., Montoya, C., Kovacs-Nagy, R., Wagner, M., … Hoefele, J. (2017). Identification of a novel heterozygous de novo 7-bp frameshift deletion in PBX1 by whole-exome sequencing causing a multi-organ syndrome including bilateral dysplastic kidneys and hypoplastic clavicles. *Frontiers in Pediatrics*, *5*(November), 1–7. https://doi.org/10.3389/fped.2017.00251

Slavotinek, A., Risolino, M., Losa, M., Cho, M. T., Monaghan, K. G., Schneidman-Duhovny, D., … Shieh, J. (2017). De novo, deleterious sequence variants that alter the transcriptional activity of the homeoprotein PBX1 are associated with intellectual disability and pleiotropic developmental defects. *Human Molecular Genetics*, *26*(24), 4849–4860. https://doi.org/10.1093/hmg/ddx363

Sun, M., Lou, J., Li, Q., Chen, J., Li, Y., Li, D., … Liu, Y. (2019). Prenatal findings and molecular cytogenetic analyses of a de novo interstitial deletion of 1q23.3 encompassing PBX1 gene. *Taiwanese Journal of Obstetrics and Gynecology*, *58*(2), 292–295. https://doi.org/10.1016/j.tjog.2019.01.022

Tanno, P. Le, Breton, J., Bidart, M., Satre, V., Harbuz, R., Ray, P. F., … Coutton, C. (2017). PBX1 haploinsufficiency leads to syndromic congenital anomalies of the kidney and urinary tract (CAKUT) in humans. *Journal of Medical Genetics*, *54*(7), 502–510. https://doi.org/10.1136/jmedgenet-2016-104435
